# Similar gene expression profiles of sporadic, *PGL2*-, and *SDHD*-linked paragangliomas suggest a common pathway to tumorigenesis

**DOI:** 10.1186/1755-8794-2-25

**Published:** 2009-05-11

**Authors:** Erik F Hensen, Jelle J Goeman, Jan Oosting, Andel GL Van der Mey, Pancras CW Hogendoorn, Cor WRJ Cremers, Peter Devilee, Cees J Cornelisse

**Affiliations:** 1 Department of Otolaryngology and Head and Neck Surgery, Leiden University Medical Center, the Netherlands; 2 Department of Pathology, Leiden University Medical Center, the Netherlands; 3 Department of Medical Statistics, Leiden University Medical Center, the Netherlands; 4 Department of Otolaryngology and Head and Neck surgery, University Medical Center St. Radboud, the Netherlands; 5 Department of Human Genetics, Leiden University Medical Center, the Netherlands

## Abstract

**Background:**

Paragangliomas of the head and neck are highly vascular and usually clinically benign tumors arising in the paraganglia of the autonomic nervous system. A significant number of cases (10–50%) are proven to be familial. Multiple genes encoding subunits of the mitochondrial succinate-dehydrogenase (SDH) complex are associated with hereditary paraganglioma: *SDHB*, *SDHC *and *SDHD*. Furthermore, a hereditary paraganglioma family has been identified with linkage to the *PGL2 *locus on 11q13. No SDH genes are known to be located in the 11q13 region, and the exact gene defect has not yet been identified in this family.

**Methods:**

We have performed a RNA expression microarray study in sporadic, *SDHD*- and *PGL2*-linked head and neck paragangliomas in order to identify potential differences in gene expression leading to tumorigenesis in these genetically defined paraganglioma subgroups. We have focused our analysis on pathways and functional gene-groups that are known to be associated with SDH function and paraganglioma tumorigenesis, i.e. metabolism, hypoxia, and angiogenesis related pathways. We also evaluated gene clusters of interest on chromosome 11 (i.e. the *PGL2 *locus on 11q13 and the imprinted region 11p15).

**Results:**

We found remarkable similarity in overall gene expression profiles of *SDHD *-linked, *PGL2*-linked and sporadic paraganglioma. The supervised analysis on pathways implicated in PGL tumor formation also did not reveal significant differences in gene expression between these paraganglioma subgroups. Moreover, we were not able to detect differences in gene-expression of chromosome 11 regions of interest (i.e. 11q23, 11q13, 11p15).

**Conclusion:**

The similarity in gene-expression profiles suggests that *PGL2*, like *SDHD*, is involved in the functionality of the SDH complex, and that tumor formation in these subgroups involves the same pathways as in SDH linked paragangliomas. We were not able to clarify the exact identity of *PGL2 *on 11q13. The lack of differential gene-expression of chromosome 11 genes might indicate that chromosome 11 loss, as demonstrated in *SDHD*-linked paragangliomas, is an important feature in the formation of paragangliomas regardless of their genetic background.

## Background

Paragangliomas are tumors originating in cells of neural crest origin in the extra-adrenal paraganglia associated with the autonomic nervous system. Most paragangliomas arise in the parasympathetic paraganglia of the head and neck region, but they can also arise in the parasympathetic paraganglia of the mediastinum or in the orthosympathetic para-aortic and retroperitoneal paraganglia. They are highly vascular and usually characterized by an indolent, non-invasive growth pattern. Most cases are sporadic, but a significant number (10–50%) have been shown to be familial. Mutations in 3 of the 4 genes encoding subunits of succinate dehydrogenase (SDH, complex II in the mitochondrial respiratory chain) have been implicated in the familial forms of the disease: *SDHB*, *SDHC*, and *SDHD *[1-3]. In our population, the majority of hereditary paraganglioma cases are associated with two founder mutations in the *SDHD *gene on 11q23[4]. In addition to these SDH related cases, another hereditary paraganglioma family has been identified with linkage to a region on 11q13, the *PGL2 *locus[5]. No mitochondrial complex II genes, including *SDHA*, are located in the 11q13 region, and the identity and function of the *PGL2 *gene are yet unknown. Mutations in *SDHB*, *SDHC *and *SDHD *are also implicated in the formation of phaeochromocytomas, tumors arising in cells derived from the neural crest in the adrenal medulla [6-8]. In *PGL2 *-linked cases no association with phaeochromocytoma formation has been found to date. A recent genome-wide expression study of phaeochromocytomas identified two distinct clusters: one containing SDH- and *VHL*-associated phaeochromocytomas and another containing *MEN2*- and *NF1*-associated phaeochromocytomas, while both clusters contained sporadic cases[9]. The cluster containing *VHL *and SDH associated phaeochromocytomas was characterized by a transcription signature of reduced oxidoreductase activity and increased angiogenesis and hypoxia[9]. In order to gain further insight into *PGL2 *function and identity, we have performed a gene expression study evaluating gene expression in head and neck paragangliomas of different genetic backgrounds: *SDHD *-linked, *PGL2 *-linked and sporadic cases without a mutation in the *SDHB*, *SDHC *or *SDHD *gene. In addition to a supervised gene-based analysis, a supervised pathway-based analysis was performed, evaluating differences in gene-expression for predefined pathways and functional gene groups. We evaluated in more detail gene groups that are known to be associated with SDH function and paraganglioma-or phaeochromocytoma formation, i.e. metabolism, cell cycle, hypoxia, and angiogenesis related pathways. In addition, we evaluated the gene sets that differentiate the SDH/*VHL *– from the *NF1*/*MEN2*-associated phaeochromocytoma cluster in the aforementioned phaeochromocytoma gene expression study, using our dataset[9]. Finally, gene clusters located within or close to the *PGL2 *locus on 11q13, the *SDHD *locus on 11q23, and the imprinted 11p15 region were assessed. The latter region has previously been implicated in *SDHD *-linked paraganglioma formation[10]. The results of both gene- and pathway- based analyses show remarkable similarity in the gene-expression profiles of *SDHD *-linked, *PGL2 *-linked and sporadic paragangliomas, suggesting that paraganglioma formation involves the same mechanisms and pathways in these paraganglioma subgroups.

## Methods

### Tumor specimens

Samples from head and neck paragangliomas were obtained from the tissue banks of the department of Pathology at the Leiden University Medical Center (LUMC) (all sporadic and *SDHD *related cases and one *PGL2 *-linked case) or the University Medical Center (UMC) St. Radboud (all but one *PGL2 *-linked cases). All specimens were handled according to the ethical guidelines, as described in the Code for Proper Secondary Use of Human Tissue in the Netherlands of the Dutch Federation of Medical Scientific Societies (FEDERA). Diagnosis of paraganglioma was confirmed by histology in all cases. All paragangliomas were carotid body tumors arising in the carotid bifurcation in the neck. No malignant paragangliomas were included in the study. Eighteen paraganglioma cases were selected: 7 cases with a known D92Y founder mutation in the *SDHD *gene, 6 cases from the family with significant linkage tot the *PGL2 *locus on 11q13, and 5 sporadic cases[5]. The latter were defined as 'sporadic' because mutation scanning of *SDHB*, *SDHC*, and *SDHD *was negative, while the family histories of these cases were negative for HN-paraganglioma or any of the other clinical stigmata that would suggest the involvement of *VHL*, *NF1 *or the *RET *gene.

### Mutation scanning

*SDHB*, *SDHC*, and *SDHD *genes were scanned for the presence of mutations at the laboratory for DNA diagnostics at the LUMC. All exonic regions of these genes were tested by direct sequencing using the Sanger method on an ABI 3177 Genetic Analyzer, starting with the exon containing the known Dutch founder mutations in *SDHD *followed by exons that had previously been found to contain pathogenic mutations in *SDHD*, *SDHB*, and *SDHC *(in that order) in the Dutch population[4,11]. If that remained negative, scanning was completed by analyzing the remainder of exons of these genes. More recently, the sporadic, mutation-negative cases were also examined by MLPA for the presence of large deletions in *SDHB*, *SDHC*, and *SDHD*[12]. MLPA was carried out with the P226 MLPA kit, containing probes for all exons and the promoter of each of these genes (27 different probes), according to the MRC Holland protocol[13].

### RNA isolation and microarray hybridization

Tissue samples were snap frozen in liquid nitrogen and stored at -70°C. An experienced pathologist (PCWH) estimated the tumor percentage of the samples. Only samples with a tumor percentage of more than 70% were included in the study. Sample preparation was performed according to the Affymetrix protocol (Affymetrix, Inc., Santa Clara, CA)[14]. In brief, 30 5 μm sections were taken from each frozen tissue sample and total RNA was extracted using Trizol (Life Technologies, Inc., Rockville, MD), and purified using RNeasy columns according to the manufacturers protocol (Qiagen, Valencia, CA). A minimum of 10 μg of total RNA was used to synthesize cDNA with the Superscript Choice system (Life Technologies, Rockville, MD). First strand cDNA synthesis was performed with T7- (dT24) oligomer primer, followed by second strand synthesis using T4 DNA polymerase. The resultant was purified using Phase Lock Gel and precipitated in ethanol. Synthesis of biotine labeled cRNA was performed using the BioArray HighYield Transcript Labeling Kit (Enzo Diagnostics, Inc., Farmingdale, NY) according to the protocol of the manufacturer. In vitro transcription (IVT) reactions took place at 37°C for 4,5 hours. The labeled cRNA was purified using RNeasy columns (Qiagen, Valencia, CA) and fragmented in fragmentation buffer at 94°C for 35 minutes. Fragmented cRNA prepared from each individual sample was then transferred to a specialized Affymetrix hybridization centre (Leiden Genome Technology Centre, LGTC). Here the samples were hybridized according to the manufacturers protocol in a concentration of 0,5 μg/μl to a human GeneChip U95A-v2 (Affymetrix), containing approximately 8500 probe sets. The data discussed in this publication have been deposited in NCBIs Gene Expression Omnibus (GEO), and are accessible through GEO Series accession number GSE12921[15].

### Sample size calculation

Sample size calculations were performed according to the method described by Pounds and Cheng[16].

### Normalization and expression analysis

Acquisition and quantification of array images was performed using the MAS software package (Affymetrix). All arrays were normalized with gcrma normalization using the R statistical software package available from Bioconductor [17-19].

### Unsupervised clustering analysis

Unsupervised two-way hierarchical clustering was performed with complete linkage and Euclidian distance metrics, using the R statistical software package available from Bioconductor[18,19].

### Supervised analysis

The R package 'Linear Models for Microarray Data' (LIMMA) was used for the assessment of differential expression of individual genes between paraganglioma subgroups[20]. Overall gene-expression differences between paraganglioma subgroups were evaluated with the 'global test' designed by J.J. Goeman using the R package 'global test' available on Bioconductor[18,19,21]. In order to evaluate subtle differences between paraganglioma subgroups, we analyzed all pathways in the Catalog of Human Gene Sets v2.0, containing 1687 gene sets, available from the Broad Institute as part of their publicly accessible Gene Set Enrichment Analysis (GSEA) software package [22,23]. Instead of the statistical method used in the GSEA software, we used the global test developed by Goeman et al., because the latter tends to have more power to detect gene sets with small effect sizes [24-26]. Specific attention was paid to the gene sets that were significantly represented in SDH-linked phaeochromocytomas in a recent gene-expression study by Dahia et al.[9]. Next, we applied the gene set that differentiated *SDHD *from *MEN2*-associated phaeochromocytomas in the aforementioned study to our data using the global test[9,21]. Furthermore, we performed a pathway based analysis using the global test on manually curated gene sets, focusing specifically on pathways involved in processes or conditions that are known or assumed to play a role in paraganglioma formation, i.e. proliferation, survival, apoptosis, cell cycle regulation, metabolism and hypoxia, based on pathways described in literature and the publicly available pathway databases KEGG and Biocarta [27-29]. In addition to the evaluation of functionally related genes we also performed the global test on some topographically related gene groups on chromosome 11, i.e. the *PGL2 *minimal haplotype on 11q13, the *SDHD *region on 11q23, and 11p15, an imprinted region that has been implicated in *SDHD *-linked paraganglioma and phaeochromocytoma formation[10,21]. In all, 264 manually curated pathways and functionally related gene sets were tested. All tests, both for genes and pathways, were corrected for multiple testing based on the false discovery rate (FDR) criterion, using the method of Benjamini and Hochberg[30].

## Results

Due to the rarity of *PGL2*-linked paragangliomas, sample sizes in this study are inevitably limited. In all, 21 samples were hybridized including 3 duplicates. Four samples (1 *SDHD*-linked sample, 2 *PGL2*-linked samples and 1 duplicate experiment) were excluded because of poor RNA or hybridization quality, leaving 15 different tumors in the analysis (5 sporadic, 6 *SDHD*-linked and 4 *PGL2*-linked samples) (Table 1).

**Table 1:** Clinicopathological characteristics and mutation status

sample	tumor	location	family history	mutation	sex	age at onset (yrs)	multiple paragangliomas
1	PGL04	CBT	*PGL2*	-	f	28	yes
2	PGL01	CBT	*PGL2*	-	f	28	yes
3	PGL02	CBT	*PGL2*	-	m	37	yes
4	PGL19	CBT	*PGL2*	-	f	32	yes
5	PGL05	CBT	*SDHD*	D92Y	m	43	yes
6	PGL06	CBT	*SDHD*	D92Y	m	47	yes
7	PGL13	CBT	*SDHD*	D92Y	f	29	yes
8	PGL14	CBT	*SDHD*	D92Y	f	45	no
9	PGL16	CBT	*SDHD*	D92Y	f	47	yes
10	PGL17	CBT	*SDHD*	D92Y	f	74	no
11	PGL10	CBT	SPOR	-	f	44	no
13	PGL12	CBT	SPOR	-	f	49	no
14	PGL15	CBT	SPOR	-	f	38	no
15	PGL23	CBT	SPOR	-	f	70	no
16	PGL20	CBT	SPOR	-	m	27	no

CBT = carotid body tumor; *PGL2 *= positive family history for *PGL2*-linked paragangliomas; *SDHD *= positive family history for *SDHD-*linked paragangliomas; SPOR = sporadic sample, negative family history of paraganglioma or phaeochromocytoma and no mutation in the *SDHB*, *SDHC *or *SDHD *gene; D92Y = Asp92Tyr, a Dutch founder mutation in the *SDHD *gene; m = male patient, f = female patient.

### Sample size calculation

Calculations showed that with this sample set and assuming that at least 30 to 35 genes are truly differentially expressed between subgroups with a fold change of 2.0 or more, at least 10 differentially expressed genes would be detected with a false discovery rate of 0.1.

### Unsupervised analysis

**Figure 1:**
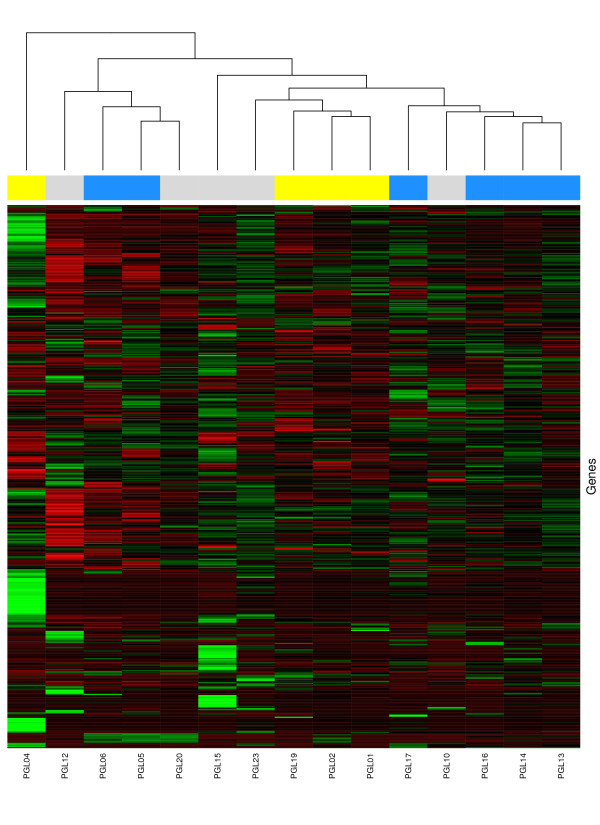
**Two way hierarchical clustering analysis of genetically defined paraganglioma subgroups**. Two way hierarchical clustering of *PGL2*-linked (yellow squares in the top row), *SDHD*-linked (blue squares in the top row), and sporadic (grey squares in the top row) head and neck paragangliomas. Samples are represented as columns and genes as rows. Expression levels are normalized for each gene. The mean is zero, and the color scale indicates the expression of the gene relative to the mean. Red indicates high expression, black indicates mean expression, and green indicates low expression levels. Overall gene expression is very similar for all samples, no well defined sample clusters can be found.

Two-way hierarchical clustering of *SDHD*-linked, *PGL2 *-linked and sporadic paragangliomas revealed no clear clusters. No grouping according to genetic background was found (fig. 1). In fact, overall gene expression was very similar in all paraganglioma samples, with high correlation coefficients for overall gene-expression between all tumors irrespective of genetic background.

### Supervised analysis

**Figure 2:**
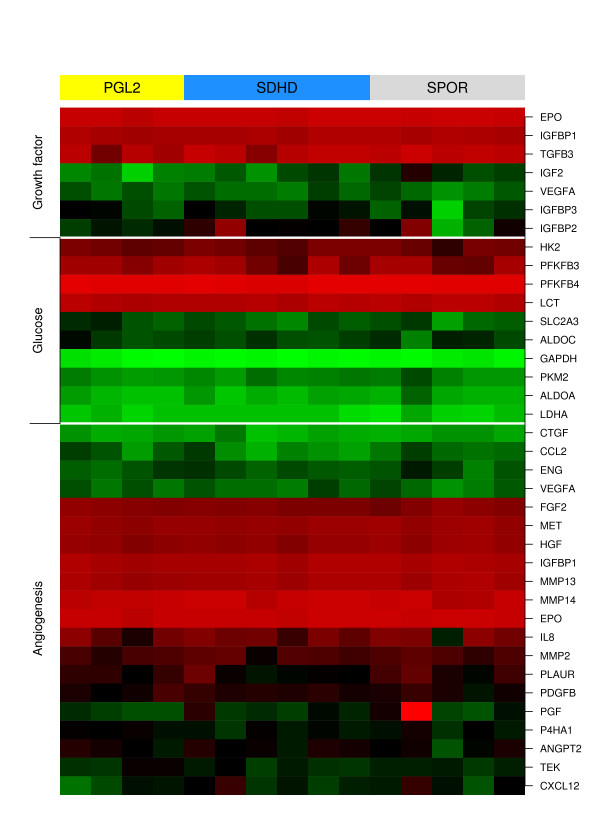
**Heatmap of *HIF1α *target genes**. Samples are represented as columns and genes as rows. Samples are ordered from left to right: *PGL2*-linked paragangliomas (yellow), *SDHD*-linked paragangliomas (blue), and sporadic paragangliomas (grey). In all, 264 pathways and functional gene sets related to processes that are assumed to play a role in paraganglioma formation (i.e. proliferation, survival, apoptosis, cell cycle regulation, metabolism and hypoxia) were tested (data not shown). None of them showed significant differential gene expression between *SDHD*-linked, *PGL2*-linked and sporadic paragangliomas, including the gene sets encoding SDH and *HIF1α *target genes involved in the processes of angiogenesis, glucose metabolism and proliferation.

**Figure 3:**
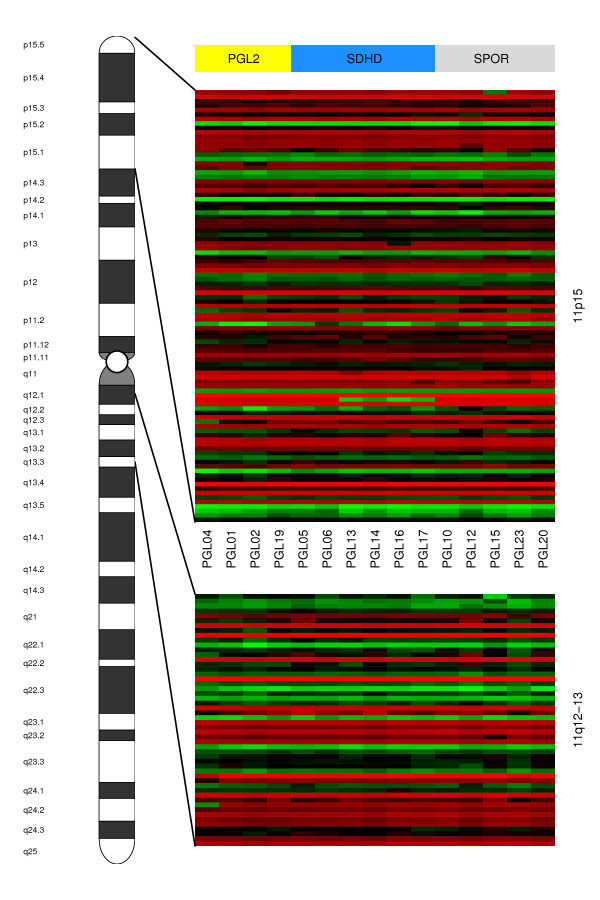
**Heatmap of chromosome 11 genes located on 11p15 and the *PGL2 *minimal haplotype on 11q13**. The upper heatmap represents genes located on chromosome 11 region 11p15, and the lower heatmap the *PGL2 *minimal haplotype located on 11q13. Samples are represented as columns and genes as rows. Samples are ordered from left to right: *PGL2*-linked paragangliomas (yellow), *SDHD*-linked paragangliomas (blue), and sporadic paragangliomas (grey). No significant differences in gene expression can be observed for genes located on the 11p15 region, which has been implicated in *SDHD*-linked paraganglioma formation, or for genes within the *PGL2 *minimal haplotype located on 11q13.

Using the LIMMA analysis, we did not find individual genes that are significantly differentially expressed between sporadic, *SDHD *and *PGL2 *-linked paragangliomas. The global test did not reveal significant differences in overall gene expression between paraganglioma subgroups. Using all 1687 functional gene sets from the Catalog of Human Gene Sets incorporated in the GSEA software, analysis with the global test revealed no significant differences in gene expression between *SDHD *and *PGL2 *-linked tumors, *SDHD *and sporadic tumors, or *PGL2 *and sporadic tumors for any gene set when corrected for multiple testing. In a recent phaeochromocytoma gene-expression study, several gene sets from the Catalog of Human Gene Sets were found to be significantly represented in SDH-associated phaeochromocytomas[9]. These gene sets comprise microtubule activity, oxidoreductase activity, *HIF1α*, angiogenesis, proteasome degradation, electron transport chain, *CCR3*, collagen and glutathione metabolism[9]. In our study, no significant differential expression between sporadic, *SDHD *and *PGL2 *-linked paragangliomas was found for these gene sets. Dahia et al. also identified a gene set differentiating SDH- from *MEN2*-associated phaeochromocytomas[9]. This gene set contained 400 probes, encoding 288 different annotated genes. 212 of these 288 genes were also represented on the Affymetrix U95A chip used in this study. No significant differential expression between sporadic, *SDHD *and *PGL2 *-linked head and neck paragangliomas was observed for this gene set (data not shown). Next, we performed the global test on manually selected pathways assumed to play a role in paraganglioma formation, i.e. proliferation-, survival-, apoptosis, cell cycle regulation-, metabolism- and hypoxia related pathways. In all, 264 pathways and functional gene sets were tested. No significant differential expression was observed for any of these gene sets between the paraganglioma subgroups (data partially shown in fig. 2). Last, we performed a more detailed evaluation of genes located on chromosome 11 loci of interest (11q23, 11q13 and 11p15). This analysis also did not reveal significant differences between paraganglioma subgroups (data partially shown in fig. 3).

## Discussion

In our gene-expression analysis of sporadic, *SDHD *– and *PGL2 *-linked paragangliomas of the head and neck, no significant differences in gene-expression profile were observed between these genetically defined paraganglioma subgroups. Instead, we found considerable similarity between *PGL2 *-, *SDHD *– and sporadic tumor samples in both unsupervised and supervised analyses (fig. 1, 2 and 3). This correlates well with the observation that sporadic as well as *SDHD *-linked and *PGL2 *linked paragangliomas of the head and neck share important clinical characteristics like the age of onset of symptoms, the indolent growth pattern, and a usually benign behaviour of the tumor, although multiple paragangliomas are less often observed in sporadic cases [31-34]. Furthermore, all head and neck paraganglioma subtypes share the typical histological architecture of the 'zellballen', groups of neoplastic chief cells surrounded by sustentacular cells[35,36].

In a recent gene-expression study by Dahia et al. of sporadic, *SDHB*-, *SDHD *-, *VHL*-, *MEN2*- and *NF1*-associated phaeochromocytomas, two phaeochromocytoma clusters were identified: a cluster containing *VHL*-, and SDH- linked tumors and another containing *MEN2*- and *NF1*-linked tumors[9]. Gene set enrichment analysis showed that microtubule activity, oxidoreductase activity, *HIF1α*, angiogenesis, proteasome degeneration, electron transport chain, chemokine *CCR3*, collagen and glutathione metabolism gene sets were significantly represented in the gene expression signature of SDH-linked phaeochromocytomas[9]. In our study, the GSEA pathway-based supervised analysis of sporadic, *PGL2 *– and *SDHD *-linked paragangliomas did not reveal significant differences between the subgroups for all GSEA gene sets, including the gene sets that characterized SDH tumors in the study by Dahia et al.[9]. The authors also identified a gene set that differentiated SDH-linked tumors from *MEN2*-linked phaeochromocytomas[9]. When applying this differentiating gene set to our dataset, significant differences in gene expression could not be found. These findings suggest that all paraganglioma subgroups in our study share the characteristics that defined the SDH-linked tumors in the study by Dahia et al., i.e. a signature of hypoxia, reduced oxidoreductase, and increased angiogenesis[9]. Further characterization of the gene expression profiles of head and neck paragangliomas would require comparison with normal paraganglionic tissue. However, due to the microscopic size of normal paraganglia and their close anatomical relations with essential nerves and blood vessels it is not feasible to acquire this in sufficient quantity and quality to reliably perform RNA-based tests such gene-expression microarrays. In the present study, more detailed analysis of manually selected pathways and functional gene sets that are assumed to play a role in paraganglioma formation, i.e. processes of metabolism, angiogenesis and hypoxia as well as proliferation, survival, apoptosis and cell cycle related pathways also did not reveal significant differential expression between sporadic, *SDHD *-linked and *PGL2 *-linked paragangliomas. A striking finding is that there is no significant differential expression of SDH genes between paraganglioma subgroups. This is in agreement with prior observations of *SDHB *suppression and enhanced expression of *SDHA *in sporadic, *SDHD *– and *PGL2 *-associated tumors[9,37]. Of further interest is the observed similar gene expression between all paraganglioma subgroups for *HIF1α *and *HIF1α *downstream target genes (fig. 2). *HIF1α *and *HIF1α *downstream target genes have been shown to be upregulated in SDH-linked tumors[9,38-40]. The mechanism of *HIF1α *induction in tumors with SDH mutations has recently been shown to be succinate accumulation resulting from loss of SDH function, leading to inhibition of HIF-α-prolyl hydroxylases and thus to elevated *HIF1α *activity[39,41]. The transcription factor *HIF1α *regulates a host of genes that are involved in proliferation and survival, angiogenesis and glucose metabolism, and the elevated *HIF1α *activity or pseudo-hypoxic drive is thought to be the basic mechanism of tumorigenesis in SDH-linked paragangliomas[39,42,43]. It has been demonstrated that in *PGL2 *– linked tumors SDH function is disrupted, as it is in *SDHD *-linked paragangliomas[37]. *PGL2 *– and *SDHD *-linked tumors also appear to share the features of increased *HIF1α *activity and upregulation of *HIF1α *targets that results from SDH inactivity[9,37,41]. These findings may hold important clues for the function of the yet unidentified *PGL2 *gene on 11q13, as a defect in the yet unidentified *PGL2 *gene seems to have consequences similar to a mutation in the *SDHD *gene. No mitochondrial complex II genes are known to be located in the 11q13 region, but the *PGL2 *gene could affect SDH function by interfering with SDH assembly, transport or insertion into the mitochondrial membrane, or encode a cofactor that is essential for proper SDH function. Alternatively, *PGL2 *gene function could be more directly associated with *HIF1α *stability and thus constitute the pseudo-hypoxic drive that leads to paraganglioma formation. We did not find significant differences in expression between paraganglioma subgroups for the *PGL2 *minimal haplotype on 11q13, and further research to clarify the exact *PGL2 *identity is currently ongoing.

Another important clinical feature shared by both *SDHD *-and *PGL2 *-linked tumors is the remarkable parent-of origin dependent inheritance of disease. Inheritance of paraganglioma occurs in an autosomal dominant way only when paternally transmitted, while no phenotype develops after maternal transmission[44,45]. Previously, we demonstrated that in *SDHD *-linked head and neck paragangliomas and phaeochromocytomas this exclusive paternal transmission of the disease is caused by consistent loss of the entire maternal chromosome 11[10]. We hypothesized that selective loss of an as yet unidentified, imprinted gene on the 11p15 region drives this selective chromosome loss, and may also be important in the formation of non- *SDHD *linked paraganglioma[10]. In line with this hypothesis, recently *H19*, a paternally imprinted gene on 11p15, has been put forward as the tumor suppressor gene responsible for the parent-of-origin dependent inheritance in *SDHD *-linked head and neck paragangliomas[46]. In the present study, supervised analysis of all chromosome 11 probe sets on the array, as well as more detailed analysis of genes on chromosome 11p15, 11q23 (location of the *SDHD *gene) and 11q13 (location of the *PGL2 *locus), did not show significant expression differences between sporadic, *PGL2 *– and *SDHD *-linked tumors (fig. 3). It is possible that this result reflects the loss of chromosome 11 in all these paraganglioma subgroups. As the relation between chromosome loss and gene expression alterations is complex, we must interpret the observed lack of gene expression differences between these groups cautiously in this context. It has been shown previously that all SDHD-linked HN-paragangliomas show loss of the entire copy of the wildtype maternal chromosome 11, and the same applies to *PGL2 *-linked paragangliomas[10]. Partial or entire chromosome 11 loss has also been observed in sporadic paragangliomas, although only in 2 out of 9 cases[47]. Chromosome 11 loss could thus be an important step in paraganglioma formation irrespective of the genetic background.

## Conclusion

In this study of sporadic, *SDHD *– and *PGL2 *-linked paragangliomas of the head and neck, we have found very similar gene-expression profiles for all three genetic subgroups. This correlates well with observations of comparable histopathology and clinical behaviour. More detailed analysis of gene sets that have previously been shown to characterize SDH linked tumors, as well as pathways known to be implicated in SDH linked paraganglioma formation, show no differential gene-expression for these paraganglioma subgroups. This suggests that a defect in the yet unidentified *PGL2 *gene, like a mutation in the *SDHD *gene, disrupts normal SDH function. Further gene-expression analysis of the *PGL2 *locus on 11q13 in this study did not reveal the *PGL2 *identity. The lack of differential gene-expression of chromosome 11 genes between the paraganglioma subgroups might further indicate that chromosome 11 loss, as demonstrated in *SDHD*-linked paragangliomas, is an important feature in the formation of a paraganglioma regardless of the genetic background.

## Competing interests

The authors declare that they have no competing interests.

## Authors' contributions

EFH participated in the design of the study, performed the microarray experiments and drafted the manuscript. JJG and JO performed the statistical analysis and helped to draft the manuscript. CWRJC and PCWH helped with designing the study and drafting the manuscript. AGLM coordinated the study and helped to draft the manuscript. CJC conceived of the study, participated in its coordination and helped to draft the manuscript. All authors read and approved the final manuscript.

## Pre-publication history

The pre-publication history for this paper can be accessed here:


